# Contour device implantation versus coil embolization for treatment of narrow neck intracranial aneurysms

**DOI:** 10.1038/s41598-023-31877-1

**Published:** 2023-03-25

**Authors:** Karim Mostafa, Fernando Bueno Neves, Friederike Gärtner, Sönke Peters, Johannes Hensler, Naomi Larsen, Tristan Klintz, Justus Mahnke, Olav Jansen, Fritz Wodarg

**Affiliations:** Department for Radiology and Neuroradiology, Universityhospital Schleswig Holstein (UKSH), Kiel University, Campus Kiel, Arnold-Heller-Street 3, Building D, 24105 Kiel, Germany

**Keywords:** Vascular diseases, Aneurysm, Cerebrovascular disorders

## Abstract

The novel Contour device is an intrasaccular flow disruption device designed for treatment of intracranial wide-neck bifurcation aneurysms. Outside its original purpose, Contour implantation can be used to treat aneurysms with a higher dome-to-neck ratio which would be suitable for conventional unassisted coil embolization. We compared both techniques in a retrospective single-center analysis. A total of 42 aneurysms from 42 patients with a dome-to-neck ratio of 1.6 or higher were included in this study. Data on technical success, implantation times, radiation dosages, procedural complications, reinterventions and recurrences were gathered and compared. Technical success was achieved in all cases with both techniques. Aneurysm embolization was achieved significantly faster in the Contour group compared to coiling (Overall p = 0.0002; r = 0.580; acute setting: p = 0.005, r = 0.531; elective setting: p = 0.002, r = 0.607). Significantly less radiation dosage was applied in the Contour group (Overall p = 0.002; r = 0.478; acute group p = 0.006; r = 0.552; elective group p = 0.045; r = 0.397). The number of complications was higher in the coiling group compared to the Contour group (Coiling 7/21 (33,3%); Contour 3/21 (14.3%). There was a higher rate of reinterventions in the coiling group (7.6% vs 21.4%). Outside its original intention, the Contour device seems to be a safe and fast alternative to coil embolization for the treatment of narrow-neck-aneurysms.

## Introduction

Over the last decades diverse techniques have been conceived for the endovascular treatment of intracranial aneurysms, including conventional and balloon- or stent-assisted coil embolization. Novel techniques in intra-saccular flow diversion and disruption have been devised at galloping paces, with the Woven EndoBridge (WEB-Device, Terumo, CA, USA) being the most studied device in this field^1–5^. The Contour Neurovascular System (Cerus Endovascular, Fremont, CA, USA) is one of the latest intrasaccular flow-disruption devices and it was originally intended for treatment of wide-neck bifurcation aneurysms^6^. By covering the aneurysm neck with a tight mesh, it leads to stasis and intrasaccular thrombus formation, comparable to flow-diverting stents, but without affection of the parent vessel. Only one prospective multicenter trial evaluated the safety and efficacy of the Contour device in patients with wide-necked bifurcation aneurysms until today^7^.

When comparing intracranial aneurysms, those with a high dome-to-neck ration (DTN), so called narrow-neck-aneurysms (NNA) will generally be easier to treat by an endovascular approach. Framing Coils with a diameter larger than the aneurysms neck can be placed with a low risk of dislocation and therefore often without the need for additional balloon- or stent-protection. For this reason, the need for single implant-solutions like intrasaccular flow-disruptors is lower in these aneurysms. However, coil embolization can be complex and time consuming especially in larger aneurysms and treatment alternatives are limited. Nowadays there is no clear recommendation as to which treatment method should be applied for which aneurysm.

The use of the Contour device in NNAs has not been addressed by any trial to this date. In our research, for the first time we retrospectively compared Contour implantation and conventional coiling in narrow-neck intracranial aneurysms.

## Methods

### Population

Patients who underwent Contour implantation after they presented either in an elective setting or with an acute aneurysmal rupture, with an intracranial aneurysm with a dome-to-neck ratio (DTN) higher than 1.6, herein defined as NNA, between 2018 and 2021 were retrospectively included in this study. For the coiling cohort, the last 21 patients who underwent non-stent- or balloon-assisted coiling of such a NNA were included. We further divided the patients into subgroups depending on their clinical presentation (acute or elective).

### Procedures

All procedures were performed using a triaxial setup with a 90 cm 6 French sheath (Neuron Max, Penumbra or Cerebase, Cerenovus), an intermediate catheter (Sofia EX, Microvention, Terumo, CA, USA) and a Microcatheter. For the coiling procedures a 0.0165 inch microcatheter was used (SL10, Stryker, MI, USA). Until the smaller sizes of the Contour device (5, 7 and 9 mm) were available for a 0.021 inch system, only 0.027 inch microcatheters were used (Headway 27; Microvention, Terumo, CA, USA; Phenom 27, Medtronic, Dublin, Ireland; XT 27 Stryker, MI, USA; VIA 27, Microvention, Terumo, CA, USA). For the Contour 21 we used Headway 21 (Microvention, Terumo, CA, USA) or Phenom 21 (Medtronic, Dublin, Ireland).

All procedures were performed in general anaesthesia by one or more of five board-certified interventional neuroradiologists (F.W., S.P., N.L., J.H., O.J.) on a biplanar angio-suite (Allura Xper FD20/10, Philipps, Amsterdam, The Netherlands) designated for neurovascular interventions at our institution. A neuroradiology senior consultant (F.W.) was either the leading angiographer or substantially involved in all Contour device implantations.

### Ethics and data collection

The protocol of this study was approved by the ethics committee of the Christian-Albrechts-University in Kiel. The need for informed consent was waived by the local ethics committee of the Christian-Albrechts-University in Kiel. This study was conducted in accordance with the STROBE guidelines and the ethical standards laid down in the 1964 declaration of Helsinki and its later amendments.

Patient characteristics and clinical follow-up data were collected retrospectively. The DTN was calculated for each aneurysm. For each patient, information about site and size of the treated aneurysm, technical success, implantation times for Contour device and coil implantation and radiation dose of the entire procedures in cGy/cm² were obtained. Implantation time was defined as the timeframe between the first image in the working projection to the first image after detachment of the Contour device or the last coil. Furthermore, recorded intraprocedural complications were assessed and follow-up times as well as aneurysm-related re-interventions and mortality were noted. All mentioned collected data were also compared between the acute and elective sub-groups.

### Interpretation of post-interventional outcomes and follow-up examinations

The immediate post-interventional success of coiling was evaluated using the three-grade modified Raymond-Roy-Scale (RRS) and for Contour implantation the O’Kelly-Marotta grading scale (OKM)^8,9^. However, these grading scales are not directly comparable, therefore we summarized RRS grades 1 and 2 and OKM grades A2-3, B1-3, C1-3 and D0 as “satisfying post-interventional result” and RRS grades 3a and b as well as OKM grade A1 as “residual perfusion of unclear significance” for grading of angiograms directly after the embolization procedure.

Angiographic follow-up examinations were graded according to RRS, as it describes the treatment success more clearly.

### Statistical analysis

Statistical analysis was conducted with Microsoft Excel 2021 and IBM SPSS. Descriptive assessment of our study cohort was performed. We assessed whether data in each group was approximately following a normal distribution, however, this could not be proven. Categorical variables were compared with the χ^2^-test. To compare mean intervention times and radiation dosage, the one-sided Mann–Whitney-U-Test was applied. U-, z-, p- and r-values were reported as indicated. In accordance to Cohen 1992, an r-value between 0.1 and 0.3 was classified as a “weak” effect, between 0.3 and 0.5 as an “intermediate” effect and over 0.5 as a “strong” effect^10^. We set the level of significance at an alpha = 0.05.

## Results

### Population

A total of 42 patients were included in this study, of which 21 received Contour device implantation and 21 conventional aneurysm coiling. Further, patients were divided into four sub-groups according to their presentation and treatment: 1. Elective Contour (n = 11); 2. Acute Contour (n = 10); 3. Elective Coiling (n = 8); 4. Acute Coiling (n = 13). Patient demographics and aneurysm characteristics are listed in Table 1. No statistical differences between the demographics of Contour and coiling patients could be detected. All patients treated in an acute setting presented with subarachnoid haemorrhage, in all other patients the aneurysms were incidental findings.

**Table 1:** Patient demographics and aneurysm characteristics.

	Acute Contour	Elective Contour	Overall Contour	Acute Coiling	Elective Coiling	Overall Coiling
Number of patients (female, male)	10 (7, 3)	11 (7, 4)	21 (14, 7)	13 (10, 3)	8 (6, 2)	21 (16, 5)
Mean age in years (range)	61 (47–84)	57 (31–74)	58 (31–84)	56 (49–89)	67 (54–80)	62 (49–89)
Mean DTN (range)	1.77 (1.68–3.33)	1.74 (1.6–2.96)	2.05 (1.6–3.33)	2 (1.8–3.8)	2.33 (1.86–3.40)	2.16 (1.8–3.8)

	Anterior Circulation	Posterior Circulation	Total Number of Aneurysms			
Acute contour group	7 (70%)	3 (30%)	10			
Elective contour group	10 (90.1%)	1 (9.9%)	11			
Acute coiling group	9 (69.2%)	4 (30.8%)	13			
Elective coiling group	4 (50%)	4 (50%)	8			

### Contour and coiling implantation times

Overall, mean implantation time for Contour was 29 ± 17 min and for coiling 54 ± 17 min. This difference was significant with a “strong” effect (U = 67.5; z = − 3.718; p = 0.0002; r = 0.580).

In the group of acute patients, mean implantation time was 67 ± 27 min for coiling. Contour device implantation was achieved significantly faster with a mean implantation time of 34 ± 20 min (U = 24; z = − 2.546; p = 0.005; r = 0.531). This effect was classified as “strong”.

In the group of elective patients, mean implantation time was 45 ± 15 min for coiling. Again, Contour implantation was achieved significantly faster with 25 ± 10 min (U = 13; z = − 2.560; p = 0.005; r = 0.587). This effect was classified as “strong” (Table 2).

**Table 2:** Comparison of implantation time, radiation dose and periprocedural complications and overall and in both subgroups.

	Overall Contour	Overall Coiling	p-value	r-value
Implantation time (min)	29 ± 17	54 ± 17	0.0002	0.580
Radiation dosage (cGy/cm^2^)	2576 (939–13,062)	11,440 (699–22,801)	0.002	0.478
Periprocedural complications	3 (14.3%)	7 (33,3%)	Ns	

	Acute Contour	Acute Coiling	p-value	r-value
Implantation time (min)	34 ± 20	67 ± 27	0.005	0.531
Radiation dosage (cGy/cm^2^)	14,360 ± 9531	3912 ± 3802	0.006	0.552
Periprocedural complications	3 (30%)	6 (46%)	Ns	

	Elective Contour	Elective Coiling	p-value	r-value
Implantation time (min)	25 ± 10	45 ± 15	0.002	0.607
Radiation dosage (cGy/cm^2^)	1374 ± 507	11,224 ± 8961	0.026	0.450
Periprocedural complications	0	1	Ns	

### Radiation dose

Mean radiation dose overall for the coiling cohort was 11,440 (699–22,801) cGy/cm^2^ and for the Contour cohort was 2576 (939–13,062) cGy/cm^2^. This difference was significant with a “strong” effect (U = 71; z = − 2.868; p = 0.002; r = 0.478).

In the acute groups, the mean radiation dose was 14,360 (1855–24,796) cGy/cm^2^ for aneurysm coiling and 3912 (939–13,062) cGy/cm^2^ for Contour implantation. This difference was significant (U = 17; z = − 2.469; p = 0.006; r = 0.552) and the effect was “strong”.

In the elective groups, the mean radiation dose for patients who underwent aneurysm coiling was 11,014 (699–26,785) cGy/cm^2^. Significantly less radiation dose was used during Contour device implantation with a mean of 1374 (818–2049) cGy/cm^2^. (U = 21; z =− 1.688. p = 0.045; r = 0.397). This effect was “intermediate” (Table 2).

### Immediate postinterventional aneurysm occlusion

Overall, 16/21 coiling patients and 21/21 Contour patients were associated with a satisfying postinterventional result immediately after the procedure. In the acute cases, 9/13 coiling patients showed a satisfying postinterventional results. In the elective setting 7/8 coiling patients showed a satisfying post-interventional result.

### Procedural success and periprocedural complications

Contour implantation and coiling procedures were technically successful in all patients. All complications and re-interventions are summarized in Table 3. Overall, 7/21 (33,3%) coiling patients and 3/21 (14.3%) Contour patients had a periprocedural complication.

**Table 3:** Summary of peri-interventional complications and follow-up results at latest follow-up.

	Ruptured aneurysms		Unruptured aneruysms		Overall	
	Coiling (n = 13)	Contour (n = 10)	Coiling (n = 8)	Contour (n = 11)	Coiling (n = 21)	Contour (n = 21)
Total peri-interventional complications	6	3	1	0	7	3
Thromboembolic	3	1	1	0	4	1
Intraoperative aneurysm rupture	2	1	0	0	2	1
Device related	1	1	0	0	1	1
Post-interventional vasospasms	6	1	0	0	6	1
All-cause mortality	1	2	0	0	1	2
Lost-to-follow-up	5	4	1	2	6	6
Angiographic follow-up data available	7	4	7	9	14	13
Aneurysm related reintervention	3	1	0	0	3	1
RRS 1/2	5	2	6	8	11	10
RRS 3a/3b	2	2	1	1	3	3
Mean follow-up period (months)	16.4	5.1	9.8	10.6	15.0	9.0

Six complications (46%) were noted in the acute coiling group, with three cases of thrombus formation on the coil loops, one case of aneurysm rupture and spontaneous bleeding respectively and one case of short-term flow-impairment on the parent vessel. Six patients had to undergo re-angiography for spasmolysis, with one patient suffering from spasms at the end of the coiling procedure. Of these cases, one patient with vasospasms suffered from an infarction in the corresponding vascular territory causing remaining neurological impairment (mRs > 1) at latest follow-up. One patient with severe vasospasms died in the framework of large infarctions in both hemispheres despite interventional spasmolysis.

In the acute Contour group, three patients (30%) suffered from a complication during the procedure, including one thromboembolic event, one case of spontaneous bleeding from the aneurysm dome and one case of intra- and postprocedural vasospasms. This patient and the patient with spontaneous aneurysm rupture suffered from severe neurological impairment at latest follow-up. Further, three aneurysms received additional coiling inside the Contour device to provide optimal aneurysm occlusion (Fig. 3).

In the elective coiling group, one patient underwent intraprocedural stent-retriever thrombectomy due to a thromboembolic event. At latest follow-up, this patient did not show any symptoms related to this event.

No periprocedural complications were reported in the elective Contour group.

### Follow-up and re-interventions

Overall, follow-up data is available for 14/21 coiling patients and 13/21 Contour patients. At the latest available follow-up, 11 (78.6%) of the coiled aneurysms and 10 (76.9%) of the aneurysms treated with Contour implantation were fully occluded (RRS 1/2). In both groups three aneurysms were graded as RRS3a/3b. In the available follow-up, 3/14 (21.4%) coiling patients and 1/13 (7.7%) Contour patients had to undergo reintervention due to aneurysm progression.

In the acute coiling group, angiographic follow-up was available for eight patients. One patient died and five were lost to follow-up. Mean follow-up time was 16.4 months, with the longest available data after 46 months. At latest available follow up, five aneurysms were graded as RRS 1/2 and two as RRS 3a. Three patients were in need of a reintervention due to aneurysm progression.

In the acute Contour group, angiographic follow-up was available for four patients. Two patients were graded as RRS 1 after six and 12 months respectively. One patient was graded as RRS 3a and another one as RRS 3b after 6 months. Two patients died during the hospital stay due to non-aneurysm related causes. One patient had to undergo reintervention.

In the elective coiling group, angiographic follow-up was available for seven patients. Mean follow-up time was 9.8 months with longest available data after 42 months. Six aneurysms were graded as RRS 1/2 and one as RRS 3a. No reinterventions were reported in this group.

In the elective Contour cohort, angiographic follow-up data is available for 9 patients. Two of the 11 patients in this group were lost to follow-up. Mean follow-up time was 10.7 months with longest available data after 18 months. After 6, 12 and 18 months, eight aneurysms were graded as RRS 1/2 and one as RRS 3a. No re-interventions were reported in this group.

## Discussion

The Contour Neurovascular System is a novel development in intrasaccular flow-disruption devices and adds to the interventional armamentarium for minimally invasive treatment of intracranial aneurysms. Despite the scarce literature regarding this device and its yet restricted worldwide employment, preliminary reports have supported both its efficacy and safety for the use in wide-neck-bifurcation aneurysms^6,7^. This research intended to explore the peri-interventional aspects of Contour implantation in patients with NNAs in both acute and elective settings in comparison to a matched group of aneurysms treated with unassisted coil embolization.

### Considerations of Contour implantation in narrow-neck aneurysms

**Figure 1:**
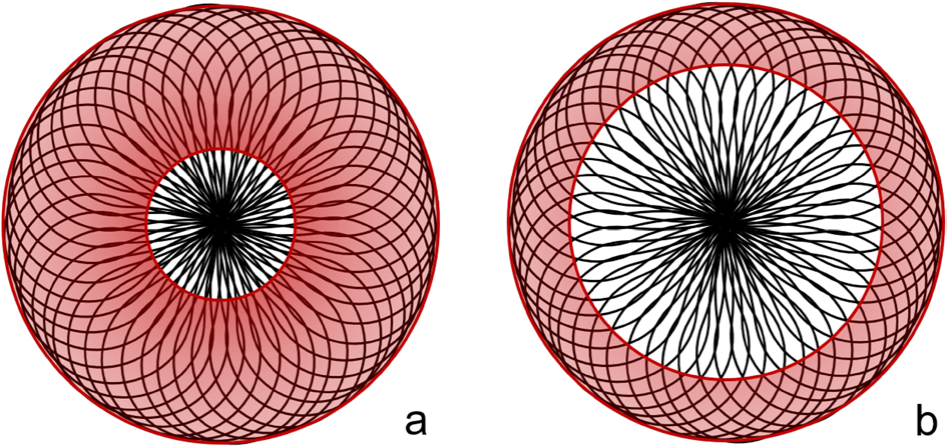
Schematic comparison of the Contour device in narrow- and wide-necked aneurysms. In both figures, the red circles depict aneurysms, with the inner circles representing the aneurysm neck and the outer circle the aneurysm dome. Image a represents (**a**) narrow-neck aneurysm and (**b**) a wide-neck aneurysm. Due to its geometry, there is a very high mesh density of the Contour device at the entry level of narrow-neck aneurysms, likely causing a favourably higher flow-disrupting effect (**a**) compared to wide-neck aneurysms (**b**).

The geometry of the Contour Neurovascular system was developed for treatment of wide-necked bifurcation aneurysms. In narrow-neck intracranial aneurysms, its design however could cause an even stronger flow-disruption effect due to two reasons: (1) the shape of the Contour device will lead to a higher mesh density and likely an amplification of flow-disruption at the entry level of narrow-neck aneurysms (Fig. 1). (2) As the device cannot stretch out to its maximum diameter at the aneurysm base, it will lay itself higher on the aneurysm wall covering more intrasaccular volume which further supports flow disruption. The strength of the latter effect however depends on the exact aneurysm geometry.

### Implantation times

The implantation of the Contour Device could be achieved significantly faster than coil implantation overall and in both sub-groups. This is a conclusive finding, as aneurysm coiling involves the insertion of multiple implants, while the Contour Neurovascular System is a single implant solution. Liebig et al. reported a mean instrumentation time of 19 (5–67) min for Contour device implantations in wide-necked bifurcation aneurysms in 34 patients, which is in good accordance to our findings^7^. The fast implantation times of the Contour Device were observed even though some of the included cases were the first ever performed at our institution, whereas all interventionalists had profound experience in coil embolization.

### Radiation dosage

In our study, we found significantly lower radiation dosages in Contour implantation overall and in both subgroups. In 2019, Forbrig et al. reported a mean dose-area product of 119 Gy/cm^2^ for coiling of 26 aneurysms and of 128 Gy/cm^2^ for Web implantation in 21 aneurysms^11^. For coiling, their result is comparable to our findings, however we had significantly less radiation exposure in the Contour groups. This was still the case even when in acute cases, angiograms of all vascular territories for exclusion of other aneurysms as well as cone-beam CT scans after ventricular drainage implantation were performed. It is evident that Contour implantation as a single implant solution can be achieved with remarkably less radiation exposure, which is in harmony with the observed faster implantation times.

### Immediate postinterventional results and postinterventional occlusion

**Figure 2:**
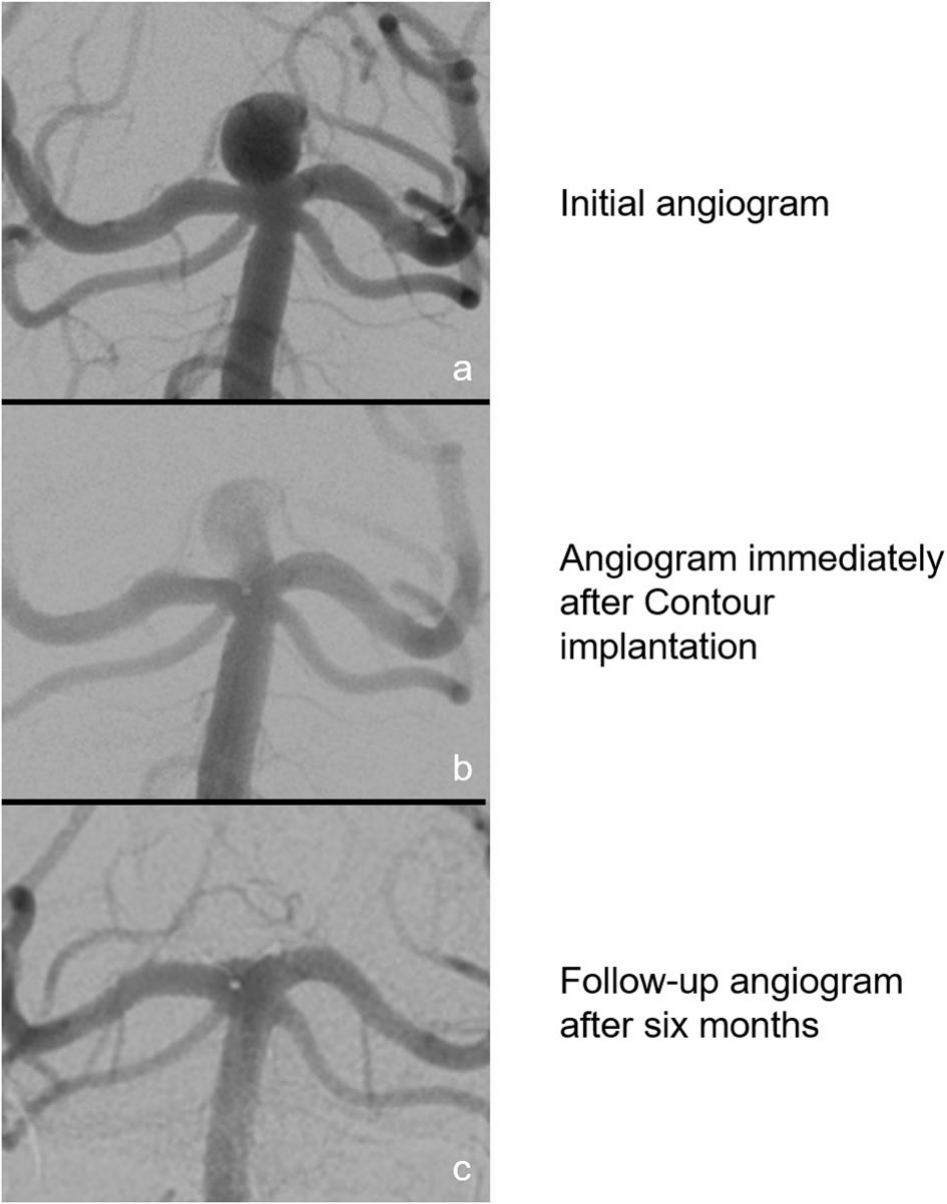
Initial, postinterventional and follow-up angiograms of a patient after Contour implantation into an aneurysm basilar apex with a dome-to-neck ratio of 2.3. The patient presented with a basilar tip aneurysm (**a**). Immediately after Contour implantation residual aneurysm perfusion can be seen (**b**). After 6 months, the aneurysm is fully occluded on DSA images (**c**).

In our analysis, all Contour patients were associated with a satisfying post-interventional result. Residual perfusion of the aneurysm immediately after implantation, quantified as OKM A2-C3, was not considered a problematic finding, since any signs of flow disruption or stasis inside the aneurysm can be interpreted as a sign of therapeutic success. In our cohort, aneurysms tended to occlude over the course of follow-up (Fig. 2). This is in good accordance to previous studies on flow-disruptive devices^7,12^**.** However, initial aneurysm perfusion without any signs of flow-disruption or stasis (OKM A1) after Contour device implantation will as of yet remain of unclear significance.

**Figure 3:**
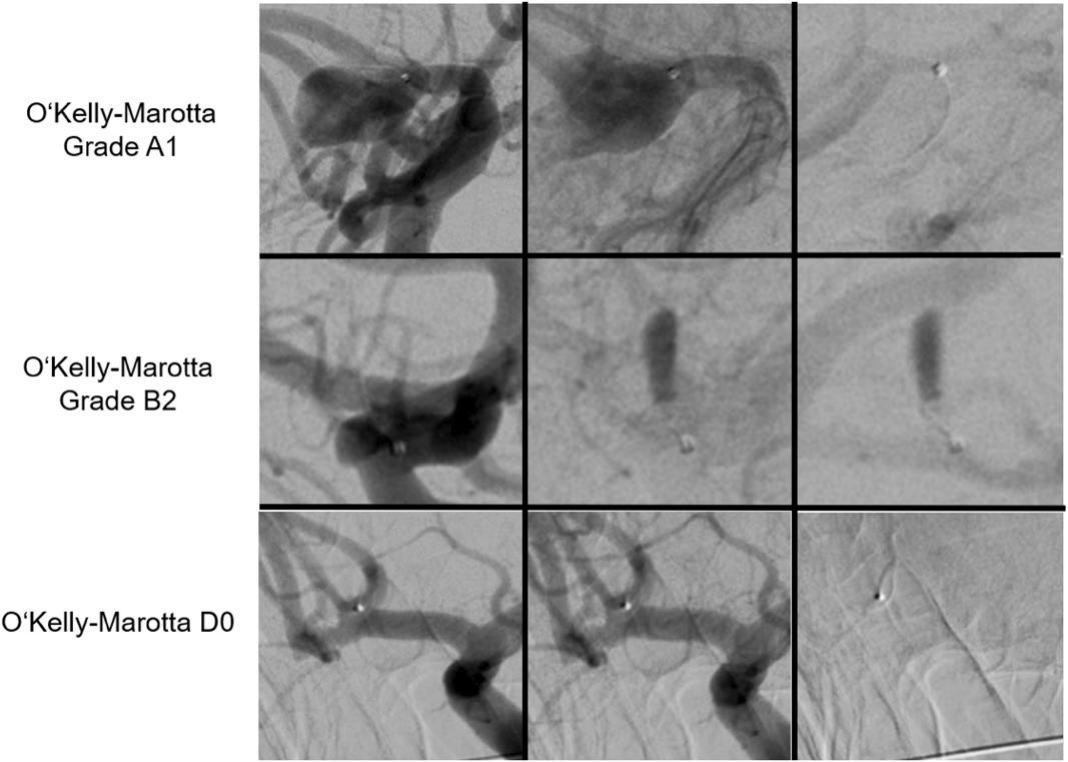
Angiograms of different outcomes in the O’Kelly Marotta grading scale immediately after Contour device implantation.

**Figure 4:**
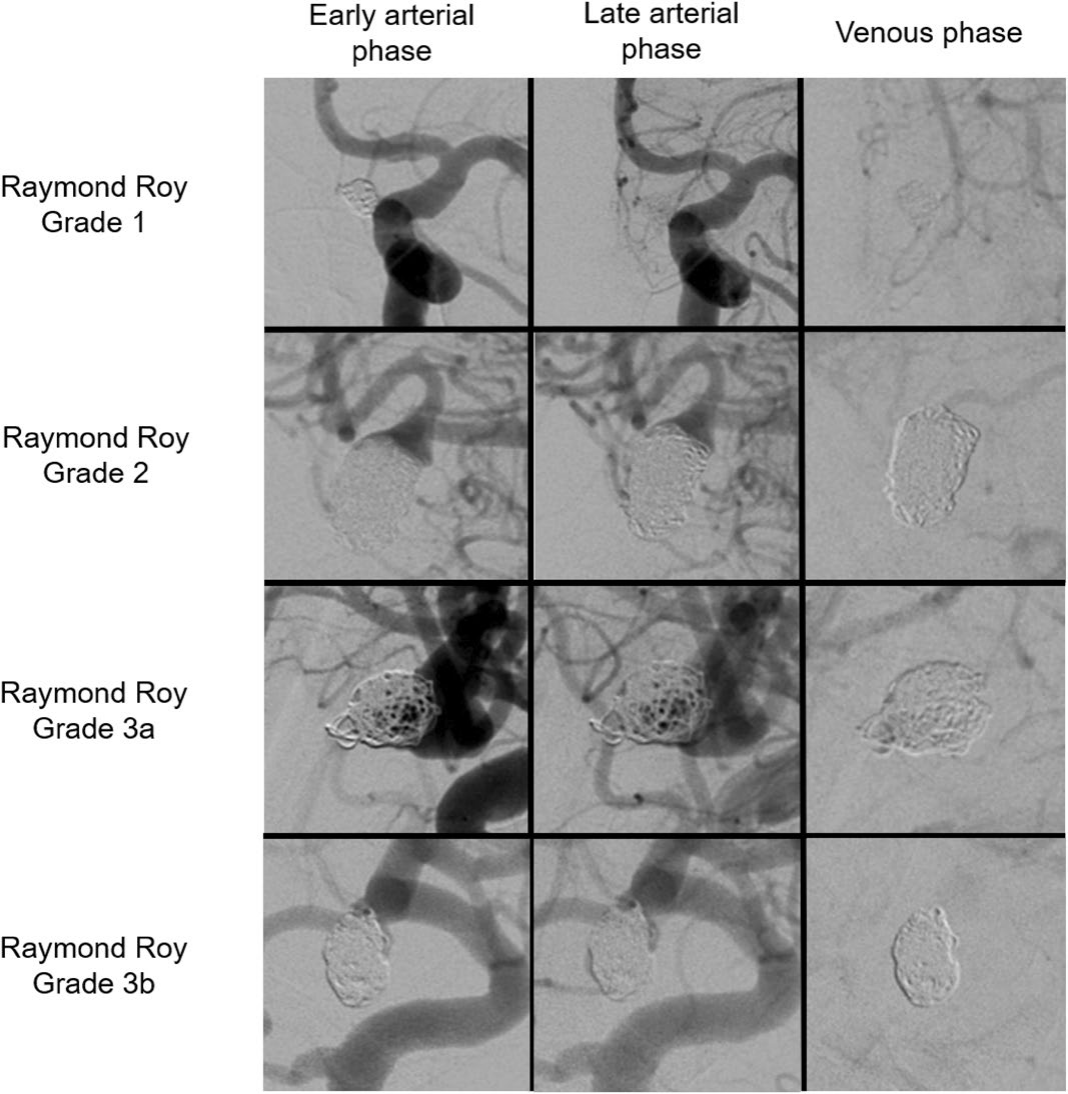
Angiograms of different outcomes in the Raymond Roy grading scale immediately after conventional aneurysm coiling.

Residual aneurysm perfusion after implantation is possibly a relevant finding when using the Contour device in the setting of acute aneurysmal rupture, as any active aneurysmal bleeding will likely only be mitigated by the device. However, with aneurysm rebleeding within 72 after initial bleeding being a rather rare event, our experience has shown that the initiation of flow stasis inside the aneurysm by Contour implantation, quantified as OKM A2-C3, is enough to provide sufficient sealing in the immediate post-interventional setting^13^. Immediate complete flow-stasis inside the aneurysm can be achieved with additional coiling inside the Contour device, which may be considered in the setting of lack of flow stasis (OKM A1, RRS 3a/b) or other reasons resulting in insufficient sealing of the aneurysm (Figs. 3 and 4)^14^.

Five aneurysms (23.8%) were instantly occluded after Contour implantation. The CERUS study reported an instant occlusion rate of 10% in thirty patients with wide-necked bifurcation aneurysms after treatment with Contour^7^. It is likely that the aforementioned properties of the Contour device when used in NNAs contributed to our higher number of instantly occluded aneurysms. This poses an interesting topic for further research.

### Periprocedural complications, reinterventions and follow-up

All complications and reinterventions are summarized in Table 3. Overall and across both subgroups no statistical differences could be found regarding complications or reinterventions. Especially in the setting of acute subarachnoid haemorrhage, peri-interventional complications were in most cases linked to the haemorrhage itself along with vasospasms and damages to the surrounding brain tissue rather than to the devices used to embolize the aneurysms^15^. However, four coiling patients had device-related complications against only one such complication in the Contour cohort. This was determined by consensus between the leading angiographers on these cases.

In the acute setting patients, we report two complications leading to neurological impairment (mRs > 1) at latest follow-up for both the Contour and coiling group. In the Contour group, one patient suffered from severe early vasospasms leading to bilateral infarctions. As for the coiling group, one patient suffered from excessive thrombus formation on the coil loops leading to infarctions.

In the Contour group, one case was associated with intraoperative aneurysm rupture while two cases were associated with it in the coiling group. Intraoperative aneurysm rupture is a high morbidity and mortality complication known to be associated with ruptured aneurysms localized in the anterior circulation, as was present in two of these cases^16,17^.

As expected, patients treated electively were associated with less periprocedural complications than those treated in an emergency setting.

Overall, there were less periprocedural complications (14.2% vs 33,3%) in the Contour group compared to the coiling group. In 2019, Liebig et al. compared conventional and balloon- or stent-assisted coiling versus WEB implantation in 67 patients and reported a complication rate of 9% for coiling and 8.9% for WEB implantation. This is considerably lower than in our findings, however they only included unruptured aneurysms in their analysis which likely is a relevant contributing factor to this low number^18^. In the regard of periprocedural complications, we conclude from our research that Contour implantation is not inferior to aneurysm coiling. Our results even suggest that Contour implantation is associated with fewer complications, however this needs to be further investigated by future research, since our cohort is too small to assume this.

In our study we found a higher rate of reinterventions in the coiling group compared to the Contour group (7.6% vs 21.4%). This is in good accordance to Liebig et al., who reported reintervention rates of 17.6% for coiling and 4.3% for WEB implantation^18^. Both their and our own results hint at possible advantageous properties of flow-disruption devices concerning reinterventions, however further research is needed to prove this hypothesis.

## Limitations

Even though being the first research to compare Contour implantation and conventional coiling in NNAs, it is valid to highlight that our small cohort due to the single-center design is not sufficient to corroborate that Contour implantation is at least as efficient as conventional coiling to treat small-neck aneurysms—our results only may suggest so and call for further research in this field. Secondly, as it may be the case worldwide, our team is notably more experienced in aneurysm coiling than in the implantation of Contour devices, which might have interfered with some results. Furthermore, even if the senior consultant was either the leading angiographer or involved in the Contour implantation procedure, it is likely that operator-dependant differences have affected the peri-interventional results.

## Conclusion

We conclude from our single-center study that the implantation of a Contour device is a feasible and faster alternative with less radiation exposure and not statistically significant periinterventional complications compared to conventional aneurysm coiling and it adds an instrument to the interventional armamentarium when treating patients with narrow-neck intracranial aneurysms.

## Data Availability

The datasets generated and analysed during the current study are available from the corresponding author on reasonable request.
